# An Association of Morphological Characteristics of Palatal Rugae and Dental Malocclusion in an Adult Indian Population: A Cross-Sectional Study

**DOI:** 10.7759/cureus.40544

**Published:** 2023-06-16

**Authors:** Bhushan Thoke, Seema Gupta, Mubasshir A Shaikh, Junaid A Syed, Vijay Sonawane, Bhushan B Patil, Manish Sharma

**Affiliations:** 1 Orthodontics, Jawahar Medical Foundation’s (JMF) Annasaheb Chudaman Patil Memorial (ACPM) Dental College, Dhule, IND; 2 Orthodontics, Saraswati-Dhanwantari Dental College & Hospital, Post Graduate & Research Institute, Parbhani, IND; 3 Orthodontics, Rural Dental College, Pravara Institute of Medical Sciences, Loni, IND; 4 Oral Pathology, Jawahar Medical Foundation’s (JMF) Annasaheb Chudaman Patil Memorial (ACPM) Dental College, Dhule, IND

**Keywords:** dental forensics, orthodontics, cast models, dental malocclusions, palatal rugae

## Abstract

Introduction

The formation of palatal rugae is completed in the early intrauterine stage, and because of their unique and permanent pattern in each individual, they can be utilized in forensics to identify a person.

Objective

The primary objective of this study was to determine an association between the rugae pattern and the dental malocclusion system described by Angle.

Materials and methods

A prospective cross-sectional, observational study was conducted on pretreatment dental casts of 400 subjects in an age range of 18-40 years. The samples were divided according to Angle’s system of classification of malocclusion into Class I, Class II, and Class III. The number, length, pattern, and orientation of the three anterior-most primary rugae on both sides of the palatal region were studied.

Results

Significant differences were noted in the mean number of palatal rugae and mean lengths of rugae 1 and 2 on the right side (p < 0.001) and rugae 3 on the left (p < 0.001) side among the different malocclusion groups. Curved and wavy patterns were predominant, and significant differences were found among the groups (p < 0.05), whereas non-significant differences were observed in the rugae orientation between the groups on the right and left sides.

Conclusions

The current study showed significant differences in the length, number, and pattern of the palatal rugae among Angle's classes of malocclusion.

Clinical implications

Palatal rugae can be effectively used to identify dental malocclusion at an early stage and can, therefore, help intercept the developing malocclusion.

## Introduction

Extensive research has been conducted on palatal rugae in numerous fields including anthropology, forensic science, and orthodontics. Substantial research has been done to assess the stability of palatal rugae [1]. However, there is still some debate regarding this fact as it has been argued that as their position is closely related to teeth, any type of tooth movement can alter their position [2]. Anatomical placement in the oral cavity affects their stability. The palatal rugae develop during the 12th to 14th week of pregnancy and retain their shape throughout life [3]. Although they are well protected inside the oral cavity from trauma and burns owing to their anatomical position, minor alterations can occur in the shape of the palatal rugae due to illness or trauma affecting the rugae in rare situations [4,5]. Palatal prints are a trustworthy and dependable tool in the field of forensic identification. Palatal rugoscopy involves studying the different forms of palatal rugae. As these rugae patterns are distinct, they can be effectively used for gender and population identification. According to twin studies, rugae patterns have a strong genetic predisposition [6].

Palatal rugae have been used in the field of orthodontics for assessing the anteroposterior movement of teeth and superimposition of dental casts, and therefore, several studies have investigated their stability with orthodontic treatment. The stability of palatal rugae and their association with Angle’s dental malocclusion can help in the early detection of malocclusion, which can lead to a favorable prognosis with minimal dentoskeletal abnormalities and reduced treatment costs and prevent the long-term detrimental impact of these malocclusions on a person's mental well-being [7]. Owing to the lack of sufficient studies, the present study was undertaken to determine whether there was any association between the pattern of palatal rugae and the system of malocclusion described by Angle. The objectives of this study were to identify the number, length, pattern, and orientation of palatal rugae in different malocclusion groups, and to determine whether the rugae pattern is sufficiently unique to be added as an additional factor for the classification for malocclusion.

## Materials and methods

A prospective, single-center, cross-sectional observational study was conducted on 400 subjects. The sample size was calculated using G power software version 3.2.9. In this study, a significance level of 0.05, a power of 95%, and an estimated sample size of 400 were used. The study was approved by the Institutional Ethics Committee (IEC) (reference number SDCRI/IEC/22/09) and followed the principles of the Declaration of Helsinki.

PECOS criteria of the study:

P: Population- Healthy subjects of age 18-40 years with full cusp class I, II, and III molar relationship

E: Exposure- Study casts of the subjects

C: Comparator- Class I, Class II, and Class III subjects were compared

O: Outcome- Study of Rugae number, length, pattern, and orientation

S: Study type: Observational study

**Figure 1:**
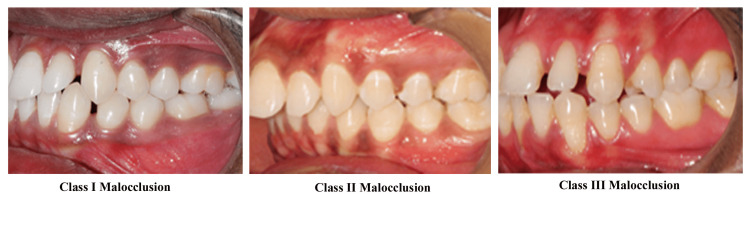
Classification of subjects according to Angle's system of malocclusion

Six hundred subjects of the local population as confirmed from their Aadhar Cards were screened from January 2022 to September 2022, who visited the Department of Orthodontics (Surendera Dental College, Sri Ganganagar, India) and 400 subjects were selected based on the following inclusion criteria: subjects with identifiable palatal rugae, no damage to the rugae pattern, subjects between the ages of 18 and 40 years with full permanent dentition, well-established relationships between the incisors and molars, and average growth pattern (FMA angle 25^0^). Subjects who underwent surgery in the palatal area or craniofacial anomalies, those with a history of thumb or finger sucking and tongue thrusting, those with a previous history or ongoing orthodontic treatment, and those with a quarter or half-cusp molar relation were excluded. The sample was divided into three groups according to Angle’s system of classification of malocclusion (Figure 1), as follows:

Group 1: Class I subjects: 136 subjects (63 males and 73 females; mean age of 24.45±6.19 years)

Group 2: Class II subjects: 136 subjects (66 males and 70 females; mean age of 26.58±5.42 years)

Group 3: Class III subjects: 128 subjects (60 males and 68 females; mean age of 25.38±7.23 years)

**Figure 2:**
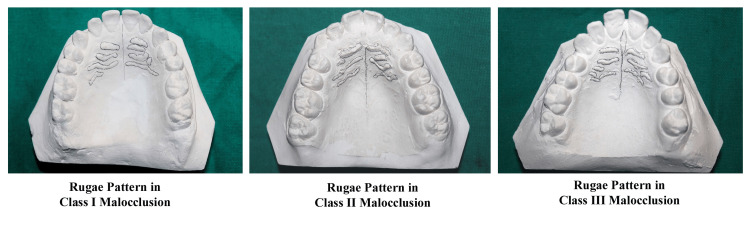
Tracing of rugae patterns in different classes of malocclusions

Before starting the study, written informed consent was obtained from all the subjects. High-quality impressions were obtained using alginate, and maxillary dental cast models were produced from high-quality plaster. Under proper lighting and magnification, the palatal rugae were traced using a sharp HB pencil (Figure 2). Digital Vernier calipers (0-150 mm ME00183, Dentaurum, Pforzheim, Germany) were used to measure linear distances after marking the palatal rugae's most medial and distal ends on the dental cast.

Assessment of the palatal rugae features

**Figure 3:**
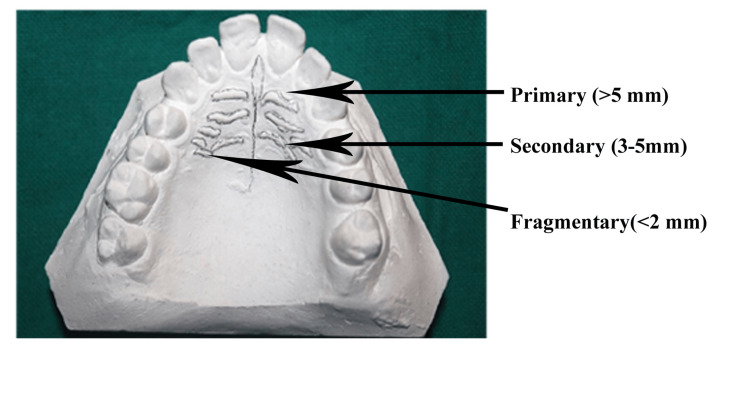
Classification of rugae (according to length) on patient's dental cast

The rugae were classified as primary (> 5 mm), secondary (3-5 mm), and fragmentary (< 2 mm). The total number of rugae on both the right and left sides was counted (Figure 3).

The three anterior-most primary rugae (labeled rugae first, second, and third) were observed for length, pattern, and orientation. The rugae were categorized in accordance with the modified Thomas and Kotze classification system to evaluate the pattern and orientation of the cast model of the maxilla [8].

Statistical analysis

All data were analyzed using IBM SPSS Statistics for Windows, Version 22 (Released 2013; IBM Corp., Armonk, New York, United States). The Kolmogorov-Smirnov test was used to test if the data were normally distributed. The Kruskal-Wallis test was applied to assess the mean differences in the number and length of the palatal rugae between the different malocclusion groups. The Chi-square test was used to compare the pattern and orientation of the palatal rugae between the groups. The principal investigator reevaluated 30 dental casts using kappa statistics for the qualitative variables and the intraclass correlation coefficient for the quantitative variables to eliminate any measurement error. Statistical significance was defined as a p-value ≤ 0.05.

## Results

The evaluation of measurement error indicated excellent agreement between the two sets of data in the estimation of rugae number and length (Tables 1, 2), whereas the pattern and orientation assessment showed moderate to substantial agreement (Table 3).

**Table 1:** Assessment of reliability of measurements for the length of primary rugae using the intraclass correlation coefficient (ICC) > 0.75 = excellent agreement; 0.4 - 0.75 = fair agreement; < 0.4 = poor agreement

Primary Rugae	Side	Reading 1 (n=30)	Reading 2 (n=30)	ICC
		Mean±SD (mm)	Mean±SD (mm)	
First	Right	6.75±1.31	6.13±1.01	0.915
	Left	7.45±1.35	7.06±1.26	0.876
Second	Right	6.91±1.42	7.11±1.45	0.931
	Left	7.51±1.42	7.01±1.37	0.929
Third	Right	7.61±1.43	7.18±1.31	0.869
	Left	7.22±1.51	7.54±1.41	0.816

**Table 2:** Assessment of reliability of measurements for the number of rugae using the intraclass correlation coefficient (ICC) > 0.75 = excellent agreement; 0.4 - 0.75 = fair agreement; < 0.4 = poor agreement

Type of Rugae	Side	Reading 1 (n=30)	Reading 2 (n=30)	ICC
		Mean±SD	Mean±SD	
Primary	Right	2.447±0.824	2.197±0.792	0.932
	Left	2.52±0.833	2.67±0.845	0.941
Secondary	Right	1.32±0.679	1.46±0.645	0.862
	Left	1.18±0.686	1.29±0.697	0.882
Fragmentary	Right	0.487±0.501	0.491±0.521	0.931
	Left	0.533±0.501	0.599±0.467	0.911

**Table 3:** Assessment of reliability of measurements for pattern and orientation of rugae using Kappa Kappa Values: 0 = no agreement; 0.01–0.20 = none to slight; 0.21-0.40= fair; 0.41– 0.60 = moderate; 0.61–0.80 = substantial; 0.81–1.00 = perfect agreement.

Palatal Rugae	Side	Kappa Value	Kappa Value
		Pattern of Rugae	Orientation of Rugae
First	Right	0.67	0.72
	Left	0.62	0.61
Second	Right	0.71	0.67
	Left	0.69	0.72
Third	Right	0.62	0.68
	Left	0.58	0.65

Statistically significant differences were observed in the number of primary and secondary rugae among the malocclusion groups, but not in the number of fragmentary rugae (Table 4).

**Table 4:** Comparison of the mean number of palatal rugae among malocclusion groups using the Kruskal-Wallis test n = 400; *p < 0.05: significant; ** p < 0.001: highly significant

Type of Rugae	Side	Class 1	Class II	Class III	p Value
		Mean±SD	Mean±SD	Mean±SD	
Primary	Right	2.880±0.872	2.000±0.808	2.460±0.503	.0001**
	Left	3.060±0.818	2.100±0.839	2.400±0.495	.0001**
Secondary	Right	1.460±0.503	1.500±0.505	1.000±0.857	.0002*
	Left	1.540±0.503	1.480±0.505	0.520±0.505	.0001**
Fragmentary	Right	0.440±0.501	0.500±0.505	0.520±0.505	0.7112
	Left	0.500± 0.505	0.600±0.495	0.500±0.505	0.5173

Primary rugae were found to be more prevalent in Class I malocclusions, followed by Class III and Class II malocclusions, while secondary rugae were virtually equally distributed between Classes I and II and were less prevalent in Class III malocclusions on both the right and left sides. The mean lengths of the primary rugae are listed in Table 5.

**Table 5:** Comparison of mean lengths of palatal rugae in mm among malocclusion groups using the Kruskal-Wallis test n = 400; *p < 0.05: significant; ** p < 0.001: highly significant

Type of Rugae	Side	Class 1	Class II	Class III	p Value
		Mean±SD (mm)	Mean±SD (mm)	Mean±SD (mm)	
First	Right	7.450±1.508	6.447±1.128	6.364±0.966	.0001**
	Left	7.528±1.388	7.353±1.341	7.478±1.353	0.8034
Second	Right	6.563±1.119	6.613±1.493	7.529±1.416	.0005*
	Left	7.807±1.469	7.205±1.350	7.506±1.414	0.1068
Third	Right	7.715±1.328	7.634±1.597	7.496±1.399	0.7451
	Left	6.507±1.447	7.615±1.380	7.549±1.457	.0001**

The mean lengths of the first and second rugae in the groups on the right side (p<0.001) and the third rugae on the left side (p <0.001) were significantly different. Curved and wavy patterns were predominant, and significant differences were found among the groups (p < 0.05); however, the results did not show any specific pattern peculiar to any malocclusion type. The distributions of the different rugae patterns are listed in Table 6.

**Table 6:** Comparison of the pattern of primary palatal rugae among malocclusion groups using the Chi-square test n = 400; *p < 0.05: significant; ** p < 0.001: highly significant

Primary Rugae	Side	Pattern of Rugae	Molar Class			Chi-Sq	p-Value
			I (n=134)	II (n=133)	III (n=133)		
First	Right	Curved	50	66	78	18.521	.0001**
		Wavy	60	58	44		
		Straight	20	7	8		
		Circular	4	2	3		
	Left	Curved	53	44	56	8.495	.0143*
		Wavy	56	68	47		
		Straight	18	15	25		
		Circular	7	6	5		
Second	Right	Curved	56	74	58	7.4	.0247*
		Wavy	62	45	55		
		Straight	14	11	16		
		Circular	2	3	4		
	Left	Curved	67	69	77	11.495	.0032*
		Wavy	41	54	41		
		Straight	21	9	12		
		Circular	5	1	3		
Third	Right	Curved	73	56	61	15.826	.0004**
		Wavy	55	61	47		
		Straight	5	12	19		
		Circular	1	4	6		
	Left	Curved	52	54	78	19.583	.0001**
		Wavy	72	71	41		
		Straight	8	5	10		
		Circular	2	2	4		

There were no significant differences in the orientation between the groups on the right and left sides for rugae 1 and rugae 2, whereas statistically significant differences were observed for rugae 3 on the left side (p = 0.05). In Class I, the third rugae on the left side were oriented more anteriorly, whereas in Class III, they were oriented more posteriorly. The distribution of rugae orientations among the malocclusion groups is shown in Table 7.

**Table 7:** Comparison of the orientation of primary palatal rugae among malocclusion groups using the Chi-square test n = 400; Chi-Square test, *p < 0.05- significant; ** p < 0.001- Highly significant.

Primary Rugae	Side	Orientation of Rugae	Molar Class n=400			Chi Sq	p-Value
			I (n=134)	II (n=133)	III (n=133)		
First	Right	Posteriorly Directed	85	88	95	4.1185	0.1234
		Horizontal	5	7	2		
		Anteriorly Directed	44	38	36		
	Left	Posteriorly Directed	89	82	86	1.872	0.3922
		Horizontal	2	4	5		
		Anteriorly Directed	43	47	42		
Second	Right	Posteriorly Directed	58	62	65	5.944	0.0512
		Horizontal	1	6	2		
		Anteriorly Directed	75	65	66		
	Left	Posteriorly Directed	64	67	78	4.482	0.1063
		Horizontal	4	3	5		
		Anteriorly Directed	66	63	50		
Third	Right	Posteriorly Directed	60	61	60	0.701	0.704
		Horizontal	4	3	2		
		Anteriorly Directed	70	69	71		
	Left	Posteriorly Directed	52	62	72	6.396	0.0408*
		Horizontal	5	4	3		
		Anteriorly Directed	77	67	58		

## Discussion

Even though forensic sciences frequently employ dental comparison, DNA analysis, and fingerprinting to prove identity, the oral cavity also plays a significant role because of the unique morphology of teeth. The use of human palatal rugae has been proposed as an alternative method for identification in some circumstances, particularly if the teeth are removed for any reason, including trauma. Palatal rugae are compared to fingerprints because they are thought to be distinct for each person and are largely constant over the course of a lifetime. Once developed, they remain stable in the same place for the duration of a person's life, changing only in length as a result of normal growth.

Numerous authors have concluded that fixed orthodontic treatment results in dental changes and occasionally bony changes but does not alter the rugae pattern, reiterating the stability and singularity of the palatal rugae [2,9]. The study of palatal rugae is referred to as rugoscopy. Palatal rugae have drawn the attention of orthodontists because of their unique pattern of orientation and value as a landmark for reference in a variety of dental treatment techniques.

This was a cross-sectional, observational, prospective, single-center study. A sample size of 400 was collected for all three groups. To reduce sample variability and enable accurate estimations of demographic characteristics, a sample size of 400 research participants aged 18-40 years was selected. The age range of 18 to 40 years was considered for this study because each person receives the final shape of their palatal rugae in adolescence, supporting the idea that growth will not modify the number, size, and shape of the rugae over time [10]. According to research done by van der Linden in 1978, the anterior rugae do not lengthen after the age of 10 [11].

While differences between the sexes may exist, the literature assessment revealed contradicting data in a number of populations. Insignificant sex differences in rugae patterns have been documented in many studies [12,13]; therefore, in the present study, sex differences were not evaluated.

In the current study, the average number of primary rugae was close to three, and secondary rugae were close to two, which is in agreement with the results of previous studies [14]. There was a significant difference in the number of primary and secondary rugae in different classes of malocclusion (p<0.05); on the right side, the Class I subjects had more primary rugae on average than the Class II and III subjects. This is in accordance with a study conducted by Fatima et al. (2018) [7], who reported similar findings. However, they conducted the study in growing individuals with an insufficient sample size for an observational study. In contrast, Kapoor et al. found that Class II division 2 had the maximum number of palatal rugae [15]. The differences in the results might be because they carried out a pilot investigation and the participants were not dispersed equally among the groups with malocclusions, unlike the current study, which was conducted on a sufficient sample size with a comparable number of individuals in each group. Significant differences were also noted in the length of primary rugae; first and second primary rugae were longer in Class I on the right side, compared to Class II and III (p<0.05).

Curved and wavy patterns were predominantly observed in all the groups. This finding is in concordance with that of a previous study by Mhatre et al. [16]. However, our results were in contrast to those reported by Kapoor et al. [15], which might be due to the limited sample size used in their investigation, which prevented the detection of differences. According to reports in the literature, the dorsal surface of the tongue is crucial in determining the pattern of palatal rugae, according to Lysell [17]. The type of malocclusion may affect the tongue position; hence, different malocclusion classes are anticipated to have varied rugae patterns. Compared to skeletal Class I malocclusion, Class II malocclusion has the tongue's tip positioned more posteriorly and its dorsal section positioned more superiorly, whereas Class III malocclusion had considerably lower posterior tongue posture. Significant rugae pattern variations have been observed among groups of the same race living in different regions, which may be because the development of palatal rugae is influenced by genetic and environmental factors. According to previous reports, there is a clear hereditary predisposition in the quantity, form, and orientation of palatal rugae [6].

On the left side, the primary palatal rugae were oriented significantly differently, with anteriorly directed third rugae in Class I and posteriorly directed third rugae in Class III. This finding was in contrast with previous studies, which reported insignificant differences in the orientation of the primary palatal rugae [18]. Because rugae are asymmetrical structures with poorly known development and establishment mechanisms, inconsistent results may be obtained. The orofacial complex has many structures that are prone to comparable epigenetic changes that may affect phenotypic development. The best methods for understanding the causes of these variations may include more molecular-level investigations.

Various methods have been used to study palatal rugae. However, each of these procedures requires an advanced tool, gadget, or software that few researchers and medical professionals have access to. The current experiment used a digital vernier caliper because it is simple to use and can be used immediately for dental casts, negating the need for cast digitization and specialized knowledge [19].

The reason for varying results found in the literature might be due to non-addressal of possible confounding factors like age and gender and insufficient sample size for an observational study. We conducted our study on a sample size of 400 subjects, with 95% power, and matched groups for age and sex. The possible confounding factors in this study were age, prior orthodontic treatment, ethnicity, and gender [2,4,14]. The present study was conducted in non-growing individuals (18-40 years), addressing age as a confounding factor. We also ensured that all groups had comparable mean ages of the subjects. All patients with prior orthodontic treatment were excluded from the study. The study was conducted on subjects from a population of the same ethnic origin, and all three groups had almost equal numbers of male and female participants. Gender differences were not evaluated in this study, due to complexity and vastness of data for evaluation, which is mentioned as a limitation of the current study.

Since forensic sciences have considered palatal rugae to be stable throughout life, it is said that after five years of age, there is no change in the rugae area of a person, and the present study demonstrated significant differences in the palatal rugae in different malocclusions; therefore, they can be effectively used to identify these malocclusions at an early age. This can help orthodontists perform preventive or interceptive orthodontic procedures in patients and prevent worsening of malocclusion, which requires corrective orthodontic treatment at later stages, taxing on the cost and time of the patients. 

The limitations of the present study were the non-evaluation of gender differences and the fact that Class II was not divided into Divisions 1 and 2. This single-center study used a manual method to trace the rugae's pattern and a single investigator studied the dental casts. The current study used digital calipers for measurement, whereas currently 3-D scanners are available with measurements done by computer software, which are more reliable. Hence, future multicenter studies are required using 3-D scanners to study rugae patterns. Future studies are also required at the molecular level to understand the reason for variations of palatal rugae in different malocclusions. 

Clinical implications

For the purpose of identifying people, rugoscopy has been a significant addition to the field of forensic science. The results of this study demonstrated a statistically significant relationship between the number, length, and pattern of rugae and angle malocclusion. Palatal rugae can also be used as the classification factor for malocclusion. In summary, palatal rugae have the potential to be an additional tool for identifying a person. Palatal rugae can be employed as a stable structure for the early detection and prognosis of various dental malocclusions for preventative and interceptive treatment, which may lessen the burden of disease, the length of treatment, and the expense of treatment.

## Conclusions

Class I had a greater number and length of primary rugae as compared to Class II and Class III. Curved and wavy patterns of the primary palatal rugae were more noticeable on both the right and left sides in all malocclusion groups. The different malocclusion groups showed that the primary palatine third rugae were more commonly anteriorly directed in Class I and posteriorly directed in Class III.
